# Sciatic Nerve Variants and the Piriformis Muscle: A Systematic Review and Meta-Analysis

**DOI:** 10.7759/cureus.11531

**Published:** 2020-11-17

**Authors:** Frideriki Poutoglidou, Maria Piagkou, Trifon Totlis, Maria Tzika, Konstantinos Natsis

**Affiliations:** 1 Orthopaedics, Aristotle University of Thessaloniki, Thessaloniki, GRC; 2 Anatomy and Surgical Anatomy, National and Kapodistrian University of Athens, Athens, GRC; 3 Orthopaedic Surgery, Aristotle University of Thessaloniki, Thessaloniki, GRC; 4 Anatomy and Surgical Anatomy, Aristotle University of Thessaloniki, Thessaloniki, GRC

**Keywords:** sciatic nerve, variation, abnormality, piriformis muscle, anatomy, anomaly

## Abstract

The present systematic review and meta-analysis provides a comprehensive assessment of the sciatic nerve (SN) variants relative to the piriformis muscle (PM) and compares those variants’ prevalence among different geographical populations with respect to gender and laterality. A database search was conducted to identify cadaveric studies pertinent to SN variants relative to the PM. A total of 44 articles were included. The typical morphological pattern (type A, with the SN passing undivided below the PM) was found to be the most common variant, with 90% pooled prevalence. SN variants were more common among East Asians, with a 31% pooled prevalence of total variants. No significant differences were established with respect to gender and laterality. In greater than 10% of the population, the SN coursed through or above piriformis. Patients’ epidemiological characteristics may predispose them to certain variants. The common peroneal nerve (CPN) is more susceptible to injury during a total hip arthroplasty or a hip arthroscopy where anomalies are encountered. As anatomical variants are commonly associated with piriformis syndrome, they should always be considered during diagnosis and treatment.

## Introduction and background

The sciatic nerve (SN), the longest and widest nerve of the human body, is formed from the L4-S3 ventral roots and normally exits the pelvis, via a single trunk, through the great sciatic foramen below the piriformis muscle (PM). The SN courses in the posterior thigh compartment and divides into the tibial and the common peroneal trunk at the popliteal fossa. The tibial and common peroneal nerves (TN and CPN) are surrounded by a common epineural sheath into the SN main trunk. However, tibial and peroneal fascicular groups are separated by a connective tissue, known as the Compton-Cruveilheir septum [1]. The SN innervates the muscles of the posterior thigh compartment and all the lower leg and foot compartments.

**Figure 1:**
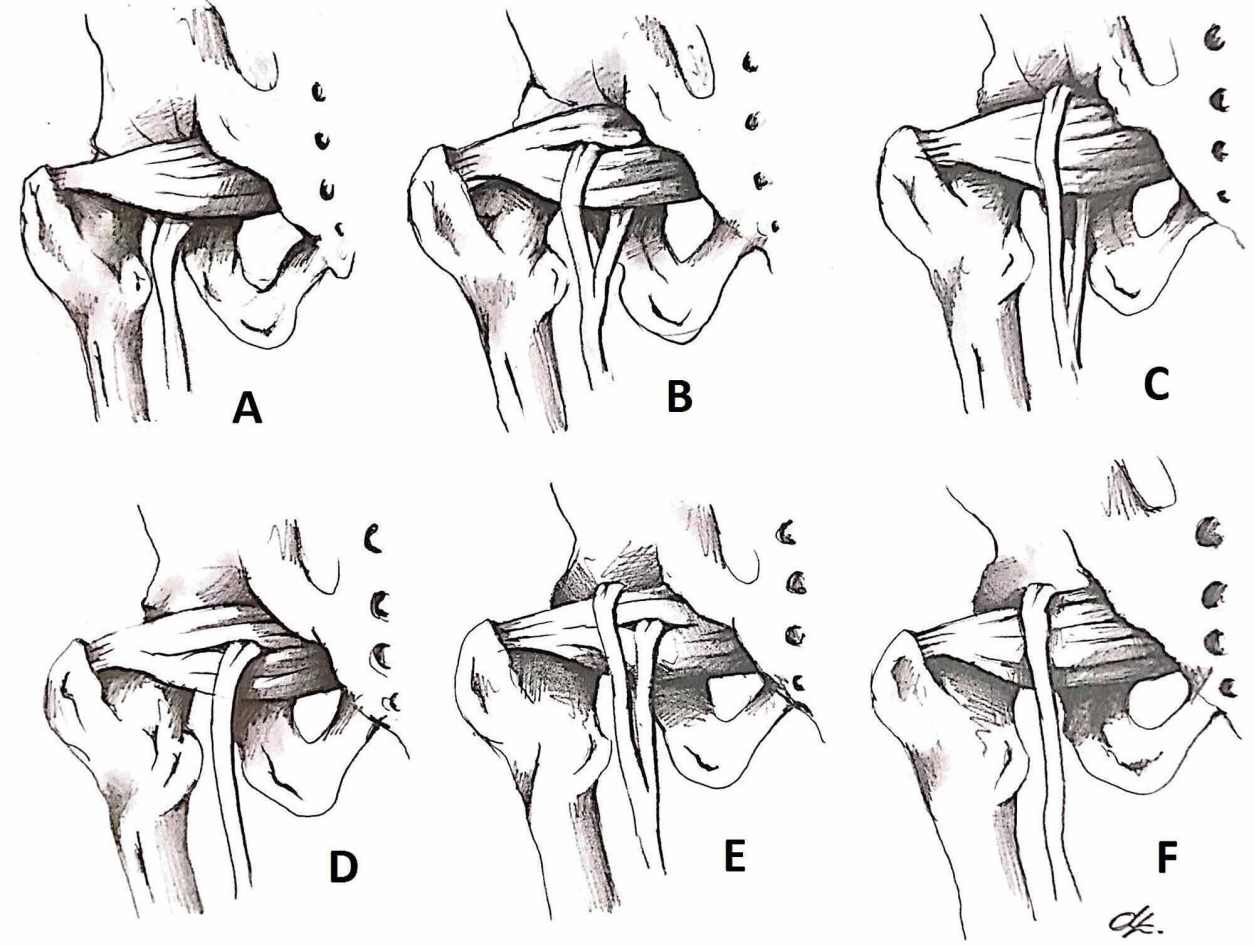
The Beaton and Anson classification system (1937)* *[3] Type A: typical morphological pattern, the SN passes below the PM undivided. Type B: the CPN exits through the PM and the TN exits below the PM. Type C: the CPN exits above the PM and the TN and below the PM. Type D: the SN exits through the PM, as a single trunk. Type E: the CPN exits above the PM and the TN through the PM. Type F: the SN passes undivided above the PM SN: sciatic nerve; PM: piriformis muscle; CPN: common peroneal nerve; TN: tibial nerve

The separate (autonomous) development of the SN tibial and peroneal divisions could explain the source of SN variants during embryonic development [2]. The possible relationships between the SN and PM were first categorized by Beaton and Anson [3] into the following six morphological types (Figure 1):

Type A: typical pattern with the SN passing below the PM, undivided

Type B: the CPN exits through the PM and TN exits below the PM

Type C: the CPN exits above the PM and TN and below the PM

Type D: the SN exits through the PM, as a single trunk

Type E: the CPN exits above the PM and TN through the PM, and

Type F: the SN passes undivided above the PM

Clinical awareness of SN variants is of high importance, as they constitute a common etiology of piriformis syndrome (a condition characterized by the SN entrapment from PM). Common symptoms include buttock pain and sciatica, which are aggravated by sitting [4]. An awareness of SN variants is crucial when performing a total hip arthroplasty, particularly via a posterior approach, SN blockade, or PM imaging-guided injections. Accurate knowledge of the typical SN anatomy and its variants could prevent a plethora of complications during procedures in the area and could aid in the diagnosis of various pathologies.

The current systematic review and meta-analysis of the literature provides an evidence-based assessment of SN variants in relation to the PM, by highlighting the variants’ prevalence among different populations, taking into account gender and laterality as well.

## Review

Methods

Search Strategy

The current systematic review and meta-analysis was conducted in compliance with the Preferred Reporting Items for Systematic Reviews and Meta-Analyses (PRISMA) statement guidelines [5]. A literature search of the databases PubMed, Scopus, SciELO, and Web of Science was conducted from their inception up to May 2020 to find studies about SN variants relative to the PM. The search terms used were as follows: “sciatic nerve”, “nervus ischiadicus”, “Ischiadic Nerve”, “Ischiatic Nerve”, “anatomy”, “variation” and “anomalies” with “AND” and “OR” as Boolean terms.

Inclusion Criteria and Study Selection

Only cadaveric studies were included. Case reports, letters to the editors, conference abstracts, and articles involving clinical or imaging studies were excluded. In compliance with the search strategy, two independent investigators screened and assessed the retrieved articles for eligibility. Any duplicates or obviously irrelevant studies were excluded. If eligibility could not be confirmed by the title or the abstract, the full text was retrieved. Reference lists of the related articles were hand-searched for any additional eligible studies in a further effort not to miss out on any relevant publications. Any disagreement regarding eligibility was resolved by a discussion between the two investigators and, if necessary, a third investigator was consulted.

Data Extraction

A dedicated data extraction form was developed for recording all relevant details, involving publication details [author(s) and year of publication], sample size, SN variants relative to the PM, gender, and laterality when recorded. In cases of nonexisting data, the authors were contacted for further clarification if possible. The classification system used was the one introduced by Beaton and Anson [3]. We exclusively analyzed our data according to types A-D, as E and F types were described as hypothetical by Beaton and Anson and most of the subsequent studies did not subcategorize their groups according to them.

Statistical Analysis

Collected data were statistically analyzed using MetaXL version 5.3 (EpiGear International, Queensland, Australia). Heterogeneity assessment was performed by using the I2 statistic and x^2^ test. I^2^ statistic of >50% and/or a p-value of <0.1 for Cochran’s Q were deemed indicators of significant heterogeneity among studies. Using the random-effects model, the weighted average and confidence intervals (95% CI) were calculated.

Results and discussion

**Figure 2:**
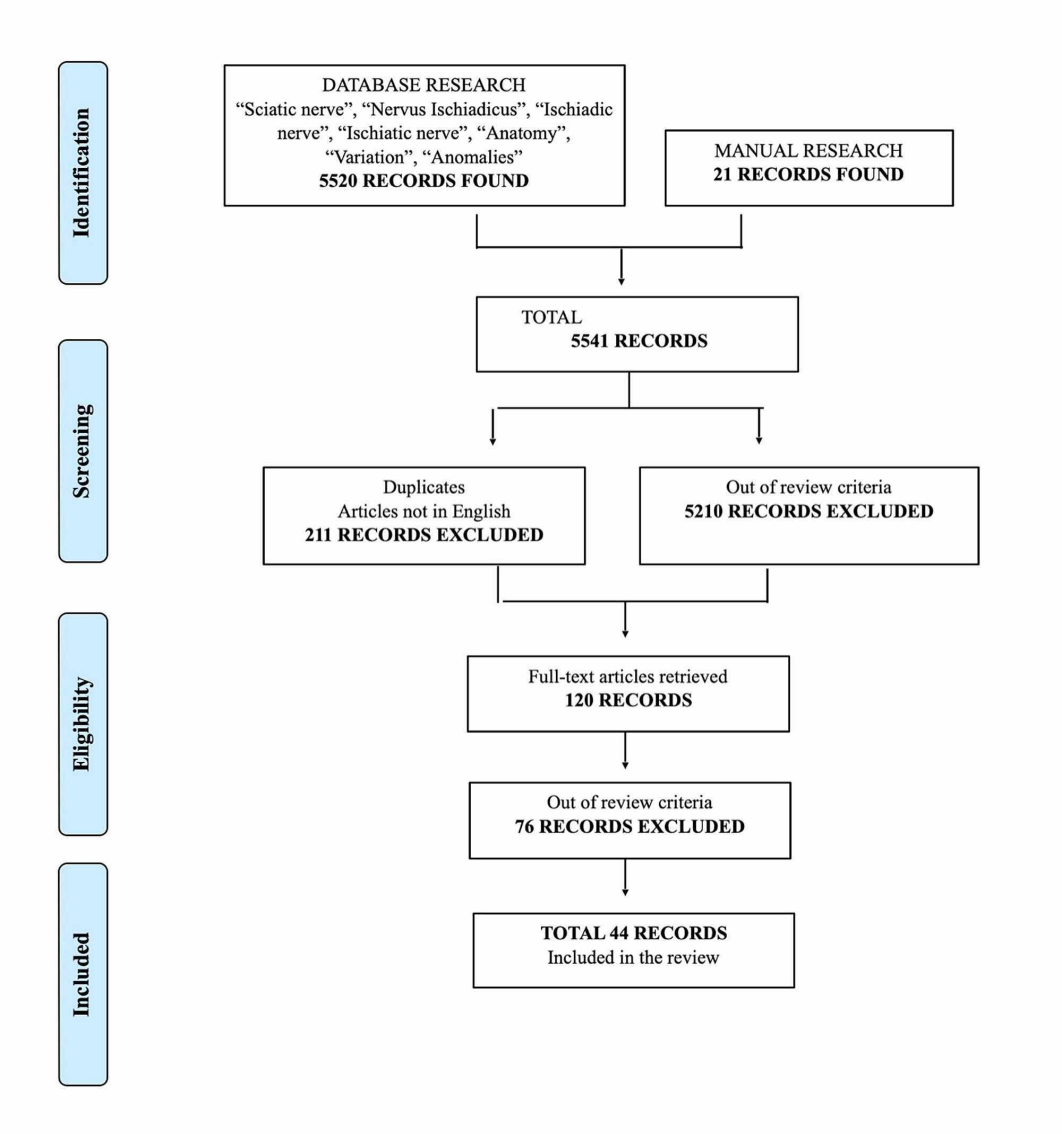
PRISMA flowchart summarizing the selection process PRISMA: Preferred Reporting Items for Systematic Reviews and Meta-Analyses

From the initial search, a total of 5,520 records were retrieved. Manual searching of reference lists yielded 21 additional articles. After exclusion of duplicates (211), articles not in English, and those irrelevant to the objectives of the present systematic review (5,210), 120 publications were retrieved in full text. Forty-four articles were deemed suitable for inclusion. The literature review selection process is summarized in Figure 2.

Study Characteristics

Study characteristics of the included articles are summarized in Table 1. A total of 44 studies (8,257 samples) were included in the systematic review and meta-analysis [3,6-48]. Included studies were published from 1893 up to 2016, were written in the English language (or at least included an abstract written in English), and involved a population origin of wide geographical distribution. Geographic subgroup analysis was based on population characteristics, the geographic location, and the number of studies derived from each country.

Prevalence of Sciatic Nerve Variants Relative to the Piriformis Muscle

**Figure 3:**
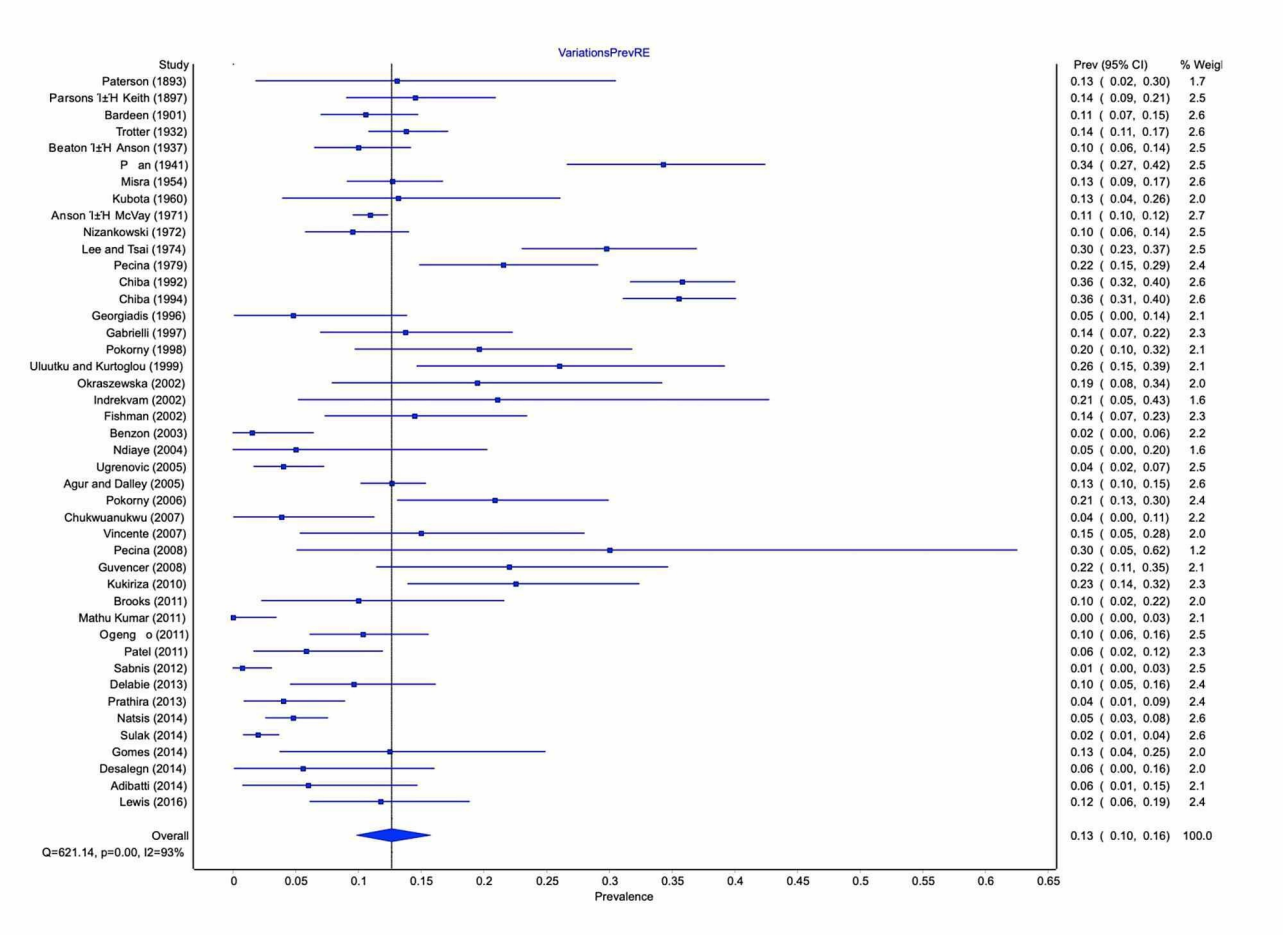
Prevalence of anatomic variations of the SN relative to the PM SN: sciatic nerve; PM: piriformis muscle

Type A was the most common morphological pattern with 90% pooled prevalence (95% CI: 83-90%) and represented the typical pattern. Total variants' pooled prevalence, including the unclassified type by Beaton and Anson [3], was 13% (95% CI: 10-16%) (Figure 3). Type B variant occurred in 8% (95% CI: 5-10%), followed by types C in 2% (95% CI: 0-3%) and D in 1% (95% CI: 0-2%).

Geographic subgroup analysis, summarized in Table 2, showed significant differences among populations. Turkey, Brazil, India, and the USA were independently analyzed since more than three studies originated from these regions, while studies derived from European, African, and East Asian countries were classified accordingly. East Asia presented the highest pooled prevalence [31% (95% CI: 26-37%)] of SN variants, followed by Turkey [14% (95% CI: 0-38%)]. In all the other regions, the upper CI limit of the variant patterns’ prevalence was less than 19%. SN variants’ distribution with respect to laterality was documented in nine studies (2,572 specimens) (Table 3). They were observed in the left side in 23% (95% CI: 16-31%), in the right side in 22% (95% CI: 13-32%), and bilaterally in 16% (95% CI: 7-26%). Only three studies (290 specimens) stated the gender of the included specimens (Table 4). Gender analysis showed a higher, but not significant, prevalence of variations in females [18% pooled prevalence (95% CI: 5-35%)] compared to males [11% (95% CI: 4-21%)].

**Table 1:** Characteristics of the cadaveric studies included in the systematic review ^a^No data reported with respect to geographic region. ^b^Data missing from one specimen. ^c^1 additional variant not described in Beaton and Anson classification. ^d^4 additional variants not described in Beaton and Anson classification: a variant with a PM with three muscle bellies and a CPN passing between superficial and intermediate muscle belly and the deep muscle belly passing through the TN; a variant in which the CPN passed between the two bellies of a double-headed PM and the TN passed below the PM; and two variants in which the SN passed below the PM and a supernumerary muscle located just superior to the PM (in the suprapiriform foramen) Note: types E and F were excluded from the meta-analysis due to the limited number of studies that included them SN: sciatic nerve; PM: piriformis muscle; CPN: common peroneal nerve; TN: tibial nerve

Author	Year of publication	Country of population origin	Sample size	Type A, n (%)	Type B, n (%)	Type C, n (%)	Type D, n (%)	Type E, n (%)	Type F, n (%)	Total variations, n (%)
Paterson [6]	1893	Scotland (Europe)	23	20 (87%)	3 (13%)	-	-	-	-	3 (13%)
Parsons and Keith [7]	1897	England (Europe)	138	118 (85.5%)	17 (12.3%)	3 (2.1%)	-	-	-	20 (14.4%)
Bardeen [8]	1901	USA	246	220 (89.4%)	25 (10.2%)	1 (0,4%)	-	-	-	26 (10.6%)
Trotter [9]	1932	USA	464	400 (86.2%)	-	-	-	-	-	64 (13.8%)
Beaton and Anson [3]	1937	USA	240	216 (90%)	17 (7%)	5 (2%)	2 (0.8%)	-	-	24 (10%)
Ming-Tzu [10]	1941	China (East Asia)	140	92 (65.7%)	46 (32.9%)	-	2 (1.4%)	-	-	48 (34.3%)
Misra [11]	1954	India	300	262 (87.3%)	18 (6%)	12 (4%)	8 (2.7%)	-	-	38 (12.6%)
Kubota et al. [12]	1960	Japan (East Asia)	38	33 (86.8%)	-	5 (13.2%)	-	-	-	5 (13.2%)
Anson and McVay [13]	1971	USA	2,008	1,789 (89.1%)	201 (10%)	13 (0.6%)	5 (0.2%)	-	-	219 (10.9%)
Nizankowski et al. [14]	1972	Poland (Europe)	200	181 (90.5%)	8 (4%)	3 (1.5%)	5 (2.5%)	3 (1.5%)	-	19 (9.5%)
Lee and Tsai [15]	1974	Taiwan (East Asia)	168	118 (70.2%)	33 (19.6%)	7 (4.2%)	3 (1.8%)	1 (1.5%)	2 (2.9%)	50 (29.8%)
Pećina [16]	1979	Croatia (Europe)	130	102 (78.5%)	27 (20.8%)	1 (0.7%)	-	-	-	28 (21.5%)
Chiba [17]	1992	Japan (East Asia)	511	328 (64.2%)	173 (33.9%)	10 (2%)	-	-	-	183 (35.8%)
Chiba et al. [18]	1994	Japan (East Asia)	442	285 (64.5%)	148 (33.5%)	9 (2%)	-	-	-	157 (35.5%)
Georgiadis et al. [19]	1996	USA	42	40 (95.2%)	2 (4.8%)	-	-	-	-	2 (4.8%)
Gabrielli et al. [20]	1997	Brazil	80	69 (86.2%)	9 (11.3%)	2 (2.5%)	-	-	-	11 (13.7%)
Pokorný et al. [21]	1998	Czech Republic (Europe)	51	41 (80.4%)	7 (13.7%)	2 (3.9%)	1 (2%)	-	-	10 (19.6%)
Uluutku and Kurtoğlu [22]	1999	Turkey	50	37 (74%)	8 (16%)	5 (10%)	-	-	-	13 (26%)
Okraszewska et al. [23]	2002	Poland (Europe)	36	29 (80.6%)	2 (5.6%)	2 (5.6%)	3 (8.3%)	-	-	7 (19.4%)
Fishman et al. [24]	2002	USA	76	65 (85.5%)	-	-	-	-	-	11 (14.5%)
Indrekvam et al. [25]	2002	Norway (Europe)	19	15 (78.9%)	-	-	-	-	-	4 (21.1%)
Benzon et al. [26]	2003	USA	66	65 (98.4%)	1	-	-	-	-	1 (1.6%)
Ndiaye et al. [27]	2004	Senegal (Africa)	20	19 (95%)	-	-	-	-	1 (5%)	1 (5%)
Agur and Dalley^a ^[28]	2005		640	557 (87%)	78 (12.2%)	3 (0.5%)	-	-	-	81 (12.7%)
Ugrenović et al. [29]	2005	Serbia-Montenegro (Europe)	200	192 (96%)	5 (2.5%)	3 (1.5%)	-	-	-	8 (4%)
Pokorný et al. [30]	2006	Czech Republic (Europe)	91	72 (79.1%)	13 (14.3%)	4 (4.4%)	2 (2.2%)	-	-	19 (20.9%)
Chukwuanukwu et al. [31]	2007	Nigeria (Africa)	52	50 (96.2%)	2 (3.8%)	-	-	-	-	2 (3.8%)
Vincente et al. [32]	2007	Brazil	40	34 (85%)	6 (15%)	-	-	-	-	6 (15%)
Pecina et al. [33]	2008	Croatia (Europe)	10	7 (70%)	3 (30%)	-	-	-	-	3 (30%)
Güvençer et al. [34]	2008	Turkey	50	38 (76%)	7 (14%)	4 (8%)		-	-	11 (24%)^b^
Kukiriza et al. [35]	2010	Uganda (Africa)	80	62 (77.5%)				-	-	18 (22,5%)
Brooks et al. [36]	2011	Brazil	40	36 (90%)	-	-	4 (10%)	-	-	4 (10%)
Muthu Kumar et al. [37]	2011	India	50	50 (100%)	-	-	-	-	-	0 (0%)
Ogeng'o et al. [38]	2011	Kenya (Africa)	164	147 (89.6%)	13 (7.9%)	4 (2.4%)	-	-	-	17 (10.4%)
Patel et al. [39]	2011	India	86	81 (94.2%)	5 (5.8%)	-	-	-	-	5 (5.8%)
Sabnis [40]	2012	India	140	139 (99.3%)	-	1 (0.7%)	-	-	-	1 (0.7%)
Delabie et al. [41]	2013	France (Europe)	104	94 (90.4%)	10 (9.6%)	-	-	-	-	10 (9.6%)
Prathiba et al. [42]	2013	India	100	92 (92%)	3 (3%)		1 (1%)	-	-	4 (4%)
Adibatti and Sangeetha [43]	2014	India	50	47 (94%)	-	-	-	-	-	3 (6%)
Desalegn and Tesfay [44]	2014	Ethiopia (Africa)	36	33 (91.7%)	2 (5.6%)	-	-	-	-	2 (5.6%)^c^
Gomes et al. [45]	2014	Brazil	40	35 (87.5%)	5 (12.5%)	-	-	-	-	5 (12.5%)
Natsis et al. [46]	2014	Greece (Europe)	294	275 (93.5%)	12 (4.1%)	1 (0.3%)	1 (0.3%)	-	1 (0.3%)	14 (4.7%)^d^
Sulak et al. [47]	2014	Turkey	400	392 (98%)	5 (1.3%)	3 (0.8%)	-	-	-	8 (1.9%)
Lewis et al. [48]	2016	USA	102	90 (88.2%)	9 (8.8%)	3 (2.9%)	-	-	-	12 (11.8%)
	Total		8,257	7,067	923	106	37	4	4	1,177
	Total prevalence (confidence interval)			90% (83-90%)	8% (5-10%)	2% (0-3%)	1% (0-2%)			13% (10-16%)
	I^2^			95%						93%
	Cochrane’s Q, p-value			0.00						0.00

**Table 2:** Subgroup analysis by geographic region

Geographic region	Type Α	Type Β	Type C	Type D	Total variations
Turkey	85% (CI: 60-100%)	9% (CI: 0-31%)	2% (CI: 0-3%)	0% (CI 0-9%)	14% (CI: 0-38%)
Europe	88% (CI: 81-91%)	9% (CI: 6-14%)	2% (CI: 0-4%)	1% (CI 0-3%)	14% (CI: 9-19%)
USA	95% (CI: 84-96%)	4% (CI: 1-9%)	1% (CI: 0-3%)	0% (CI: 0-2%)	11% (CI: 9-13%)
Brazil	89% (CI: 76-95%)	8% (CI: 2-18%)	1% (CI: 0-5%)	2% (CI: 0-6%)	13% (CI: 9-19%)
India	97% (CI: 90-99%)	2% (CI: 0-6%)	1% (CI: 0-3%)	1% (CI: 0-3%)	4% (CI: 1-9%)
East Asia	73% (CI: 59-79%)	24% (CI: 14-33%)	3% (CI: 0-7%)	1% (CI: 0-3%)	31% (CI: 26-37%)
Africa	95% (CI: 82-95%)	3% (CI: 0-8%)	1% (CI: 0-4%)	0% (CI: 0-2%)	10% (CI: 5-17%)
Total	90% (CI: 83-90%)	8% (CI: 5-10%)	2% (CI: 0-3%)	1% (CI: 0-2%)	13% (CI: 10-16%)

**Table 3:** Sciatic nerve variants relative to piriformis muscle with respect to laterality L: left; R: right; B: bilateral ^a^3 of the specimens (1 left, 2 right) had unclassified variants in Beaton and Anson classification. ^b^Data missing from 3 specimens

Author(s) (publication year)	Side (left, right)	Type A			Type Β			Type C			TypeD			Total			
		L	R	B	L	R	B	L	R	B	L	R	B	L	R	B	Total
Parsons and Keith [7] (1897)	138 (69L, 69R)	58	60	-	9	8	-	-	-	-	2	1	-	11, 15.9%	9, 13%	-	20, 14.5%
Ming-Tzu (1941) [10]	140 (70L, 70R)	45	47	36	24	22	13	-	-	-	1	1	-	25, 35.7%	23, 32.9%	13	48, 34.2%
Nizankowski et al.^α ^(1972) [14]	200 (99L, 101R)	88	93	-	5	3	-	-	3	-	4	1	-	11, 11.1%	8, 7.9%	-	19, 9.5%
Chiba (1992) [17]	511 (254L, 254R)^b^	170	157	126	78	93	37	6	4	2	-	-	-	84	100	39	183
Chiba et al. (1994) [18]	442 (221L, 221R)	148	137	113	68	80	35	5	4	2	-	-	-	73, 33%	84, 38%	37	157, 35.5%
Pokorný et al. (1998) [21]	51 (28L, 23R)	21	20	-	4	3	-	2	-	-	1	-	-	7, 25%	3, 13%	-	10, 19.6%
Uluutku and Kurtoğlu (1999) [22]	50 (25L, 25R)	18	19	-	4	4	-	3	2	-	-	-	-	7, 28%	6, 24%	-	13, 26%
Vincente et al. (2007) [32]	40 (20L, 20R)	17	17	17	3	3	3	-	-	-	-	-	-	3, 15%	3, 15%	3	6, 15%
Gomes et al. (2014) [45]	40 (20L, 20R)	18	17	17	2	3	2	-	-	-	-	-	-	2, 10%	3, 15%	2	5, 12.5%
Total	2,572	583	567	309	214	224	100	16	14	4	7	3	-	283	290	124	573
Total prevalence (confidence interval)		77% (67-85)	78% (67-88)	62% (48-74)	19% (12-28)	19% (10-30)	15% (7-26)	2% (0-6)	2% (0-6)	1% (0-4)	2% (0-5)	1% (0-4)	0% (0-3)	23% (16-31)	22% (13-32)	16% (7-26)	
I^2^		85%	91%	87%	85%	91%	87%	85%	91%	87%	85%	91%	87%	81%	89%	87%	
Cochrane’s Q, p-value		0.00	0.00	0.00	0.00	0.00	0.00	0.00	0.00	0.00	0.00	0.00	0.00	0.00	0.00	0.00	

**Table 4:** Sciatic nerve variants relative to piriformis muscle with respect to gender M: males; F: females ^a^3 of the specimens (1 male, 2 females) had unclassified variants in Beaton and Anson classification

Author (year of publication)	Number of samples (male, female)	Type A		Type Β		Type C		Type D		Total		
		M	F	M	F	M	F	M	F	M	F	Total
Nizankowski et al.^α^ (1972) [14]	200 (109M, 91F)	99, 90%	82, 91.1%	4, 3.63%	4, 4.4%	2, 1.81%	1, 1.1%	2, 1.81%	3, 3.33%	8, 7.3%	8, 8.8%	19, 9.5%
Uluutku and Kurtoğlu (1999) [22]	50 (14M, 36F)	8, 57.14%	29, 80.55%	5, 35.71%	3, 8.33%	1, 7.14%	4, 11.1%	-	-	6, 42.8%	7, 19.4%	13, 26%
Gomes et al. (2014) [45]	40 (34M, 6F)	29, 85.29%	6, 100%	-	5, 83.3%	-	-	-	-	5, 14.7%	0, 0%	5, 12.5%
Total	290 (157M, 133F)	136, 86.62%	117, 88.96%	9, 5.73%	12, 9.02%	3, 1.91%	5, 3.76%	2, 1.27%	3, 2.25%	14, 8.91%	20, 15.3%	37, 12.75%
Total prevalence (confidence interval)		87% (76-98%)	82% (59-97%)	6% (0-16%)	13% (0-34%)	3% (0-14%)	5% (0-14%)	2% (0-10%)	2% (0-9%)	11% (4-21%)	18% (5-35%)	
I^2^		63%	84%	63%	84%	84%	63%	84%	63%	37%	75%	
Cochrane’s Q, p-value		0.07	0.00	0.07	0.00	0.00	0.07	0.00	0.07	0.2	0.02	

Discussion

The present systematic review and meta-analysis provides a comprehensive and evidence-based assessment of SN variants in relation to PM. Although typical (type A) morphological pattern was the most common one (90% prevalence), its presence widely varied (64.5-100%) among the selected studies. The variant type B had 8% prevalence, followed by types C and D with 2% and 1% prevalence, respectively. Type B had a significantly higher prevalence in East Asia (24% prevalence) compared to Europe (9%), the USA (4%), and Africa (3%). Concerning gender impact, females appeared to have a higher, but not significant, prevalence of SN variants compared to males. Type B variant was twice as prevalent in females (13% prevalence) compared to males (6%). This finding could be explained by the SN's close proximity to female reproductive organs. Thus, patients’ epidemiological characteristics may predispose them to certain variants. Analysis based on laterality revealed symmetry in typical SN anatomy (62% prevalence), as well as in variant patterns' occurrence (16% prevalence).

An awareness of SN variants is essential to avoid iatrogenic nerve injury [41-44,48]. The two most common mechanisms of nerve injury, intraoperatively, are stretching and direct injury (compression or laceration) [41-43]. The SN is subject to traction forces during total hip arthroplasty, especially when performed via a posterior approach [29,30]. Therefore, SN variants relative to the PM increase the intraoperative risk of injury, either due to improper Hohmann retractor placement or by direct injury when a PM tenotomy is required [45]. The CPN is more susceptible to injury by traction when the variant types B and C are encountered, either during hip dislocation or when the lengthening of the extremity occurs [45]. The SN may also be injured after traumatic posterior hip dislocation [45], and in such cases, the coexistence of variants in the area increases the risk of injury.

Hip arthroscopy and specifically the posterolateral portal placement (as close as 11 mm to the SN) may injure the SN due to its close proximity. Type B variant may put the SN at an increased risk of injury during hip arthroscopy. Moreover, knowledge of SN variants is necessary when the SN blockade is conducted. There is a high probability of anesthetizing only the CPN or the TN when an SN high bifurcation is present, as in types B and C [39].

The piriformis syndrome is characterized by sciatic clinical manifestations caused by extrapelvic SN compression at the hip. An incidence of 6% of piriformis syndrome has been reported in patients suffering from sciatica [23,34]. Typical clinical manifestations include buttock pain with or without radiation to the ipsilateral posterior thigh and the occasional extension below the knee [4]. Pain is exacerbated by flexion, adduction, and internal rotation of the hip. Aberrations of the SN course may contribute to its compression. Pećina has suggested that type B variant (CPN course through the PM) is more commonly associated with piriformis syndrome, and especially when the CPN passes between PM tendinous parts [16]. The clinician should consider the SN variants when treating a patient with sciatica and especially when dealing intraoperatively with a piriformis syndrome [24,26].

Multiple imaging modalities are available for SN variant identification. Among them, MRI remains the gold standard. Magnetic resonance neurography can reliably and effectively identify the presence of an SN variant or even SN compression in piriformis syndrome [32,33].

Study limitations

This study has some limitations. Many studies we looked into had modified the classification system proposed by Beaton and Anson or had included variants that were stated as “non-classified”. High heterogeneity was observed among the studies, which could not be explained by geographic or gender differences alone.

## Conclusions

Based on our findings, type A (a single SN trunk coursing below the PM) is the most common morphological type and is considered as the typical pattern. SN variants are fairly common, particularly among East Asians. Clinicians should always bear in mind those variants when performing hip interventions, nerve blockade in the area, and during diagnosis and treatment of piriformis syndrome. Future clinical investigations are necessary to further evaluate SN atypical course clinical implications in relation to the PM.
